# Lymphoid Aggregates in Canine Cutaneous and Subcutaneous Sarcomas: Immunohistochemical and Gene Expression Evidence for Tertiary Lymphoid Structures

**DOI:** 10.1111/vco.13020

**Published:** 2024-10-27

**Authors:** Kristin Marie Rugh, Laura Vary Ashton, Paula Andrea Schaffer, Christine Swardson Olver

**Affiliations:** ^1^ Colorado State University Fort Collins USA

**Keywords:** B cell, dog, immunology, lymphocyte, sarcoma, T cell

## Abstract

Canine cutaneous/subcutaneous soft‐tissue sarcomas (STS) are diversely derived mesenchymal neoplasms with a risk of recurrence and/or metastasis depending on the extent of surgical excision and histologic grade. Lymphoid aggregates (LAs) are often described in these tumours but not characterised. In humans, LA characterised as tertiary lymphoid structures (TLSs) improve the prognosis of many tumours, including sarcomas. We sought to determine if LA meeting a size criterion (> 700 cells) in canine sarcomas met the criteria of TLS and the overall prevalence of LA of any size. RNA expression in large LAs versus aggregate‐adjacent sarcoma tissue (AAS) was measured in laser capture microdissected tissue and compared to curl‐derived RNA from aggregate‐free sarcomas and lymph nodes. CD3, CD20, MUM‐1 and PNAd expressions were measured using immunohistochemistry. CD20 and CD3 mRNA were more highly expressed in LA versus AAS (13.8 fold, *p* = 0.0003 and 2.3 fold, *p* = 0.043). This was supported by the IHC findings. The large LAs were also enriched in chemokine RNA expression characteristic of TLS (CXCR5 5.8 fold, *p* < 00001, CCL19 3.68 fold, *p* = 0.0209, CCL21 6.87 fold, *p* = 0.0209 and CXCL13 2.68 fold, *p* = 0.0924). Plasma cells and high endothelial venules were identified in LA containing tumours but not in control tissue. Large LAs were present in 12% of tumours, and LA of any size in 30%. We conclude that large LAs in canine STS are consistent with TLS.

## 1 Introduction

Canine cutaneous and subcutaneous soft‐tissue sarcomas (STSs) are a group of mesenchymal neoplasms that include malignant peripheral nerve sheath tumours, hemangiopericytomas, liposarcomas, fibrosarcomas, myxosarcomas, perivascular wall tumours and undifferentiated sarcomas, although there is a proposal to rename this group mesenchymal soft‐tissue tumours [1, 2]. Prognosis can be excellent with complete excision, but if marginally excised depends on histologic grade; grade 3 tumours have the highest percentage of recurrence [1, 3]. Complete excision may be challenging, as tumours may dissect into the surrounding tissues which may require a wide resection to remove. Metastasis (regional lymph node and or distant organs) is less common but occurs in marginally resected tumours with a rate of 7%–13% for Grade I, 7%–33% for Grade II and 22%–44% for Grade III (depending on the study) [4, 5, 6, 7, 8].

Human cutaneous and subcutaneous sarcomas are also variable in their histologic origins, and prognosis is dependent on the extent of surgical excision and grade [9, 10, 11, 12]. Immunotherapy has been investigated as a treatment for recurrent or advanced sarcoma as part of the PEMBROSARC clinical trial [13, 14]. Analysis of response to immunotherapy in this clinical trial shows that prognosis is improved if the tumours contain tertiary lymphoid structures (TLSs). TLSs are transient microenvironmental aggregates of B cells, plasma cells and T cells with follicular dendritic cells and high endothelial venules (HEVs) that resemble the aggregates in secondary lymphoid organs such as the lymph node and spleen [15]. They occur in areas of chronic inflammation such as sites of chronic infection, autoimmune inflammation and cancer. In human cancers, TLSs can occur in the peritumoral area or within the tumour and are thought to be capable of inducing a local immune response to tumour neoantigens [16]. TLSs have been an intense area of study in human oncology since 2008 [17]. In all tumours, they are categorised as immature (iTLS) or mature (mTLS), where mTLSs can produce an antitumour immune response and are associated with a better prognosis. Importantly, B cells are confined to the immune aggregates, whereas T cells may also be diffusely distributed throughout the tumours [18]. Although the exact definition and minimum requirements for TLS vary, human mTLSs are currently defined as containing CD4 + PD − 1 + CXCL13+ T follicular helper cells, plasma cells, BCL6+ germinal centre B cells and PNAd+ HEVs. Both iTLS and mTLS contain follicular dendritic cells [10]. For the purposes of this article, we will define a TLS‐like structure to be CD3‐, CD20‐, MUM1‐ and MECA79‐positive.

In human oncology, tumours with TLS are associated with a better prognosis than those without, especially in conjunction with immunotherapy in nonsmall cell lung carcinoma, colorectal carcinoma, pancreatic ductal adenocarcinoma, ovarian carcinoma and sarcoma [1, 2, 13, 14, 16, 17, 19, 20, 21]. Reports of TLS in veterinary oncology are rare. The possibility of TLS has been explored in one report in 2022, in canine mammary neoplasia [22]. The authors of this study immunohistochemically identified aggregates of T and B cells, follicular dendritic cells and HEVs in high‐grade mammary tumours, although their effect on prognosis was not investigated. CD3 lymphocyte–rich aggregates have been described peritumorally in feline sarcomas [23] without investigation of prognosis. We sought to characterise the composition of lymphoid aggregates (LAs) in canine sarcomas that were most likely to be comparable to TLS in humans. As such we used the definition in Fridman et al. [18], including 700 cells per aggregate, as a subset to ensure that we had all the elements to find TLS. We first sought to determine whether this subset of the LAs, specifically chosen for their organisation, fit the definition of TLS by measuring RNA and immune marker expression. We then determined the prevalence of at least one LA, which could be considered mature or immature, to gain a sense of all LAs presence in a subset of cutaneous/subcutaneous sarcomas in dogs. This includes a more general definition of aggregate of 50 cells, the reason being we wanted to gain a sense of the breadth of lymphoid presence in sarcomas. Our hypothesis was that the large organised LAs in canine sarcomas had characteristics that qualify them as TLSs.

## 2 Materials and Methods

### 2.1 Cell Line Validation Statement

No cell lines were used in this study.

### 2.2 Prevalence of Lymphoid Aggregates in STS, Case Selection and Evaluation

We desired to determine the prevalence of small LAs and large LAs in canine subcutaneous sarcomas. We defined an aggregate to be at least 50 closely associated (10 μm or less apart) lymphoid cells [18]. We also determined the presence of LAs that were 700 cells or greater. To determine prevalence, cases with a diagnosis of “sarcoma” were found by searching the electronic medical records of canine patients seen between the years 2019 and 2022 at the Colorado State University Veterinary Diagnostic Laboratory. Cases were randomly chosen from a spreadsheet until 100 total cases meeting the inclusion criteria were collected. Cases with a diagnosis of hemangiosarcoma or histiocytic sarcoma, and noncutaneous/subcutaneous sarcomas were excluded. Rhabdomyosarcomas and leiomyosarcomas as well as plexus‐associated peripheral nerve sheath tumours were exclused. Cases that also had a second malignancy diagnosed were excluded. All haematoxylin and eosin‐stained sections of each accession were digitally scanned (Olympus VS120, Olympus) and reviewed by a board‐certified pathologist (CSO) to exclude sections not containing tumour and include all sections containing tumour. We included 269 sections of the 100 tumours. Prevalence was calculated as the total number of tumours containing at least one aggregate in one section divided by the total number of tumours evaluated.

### 2.3 Case Selection for Molecular and Immunohistochemical Studies

**FIGURE 1:**
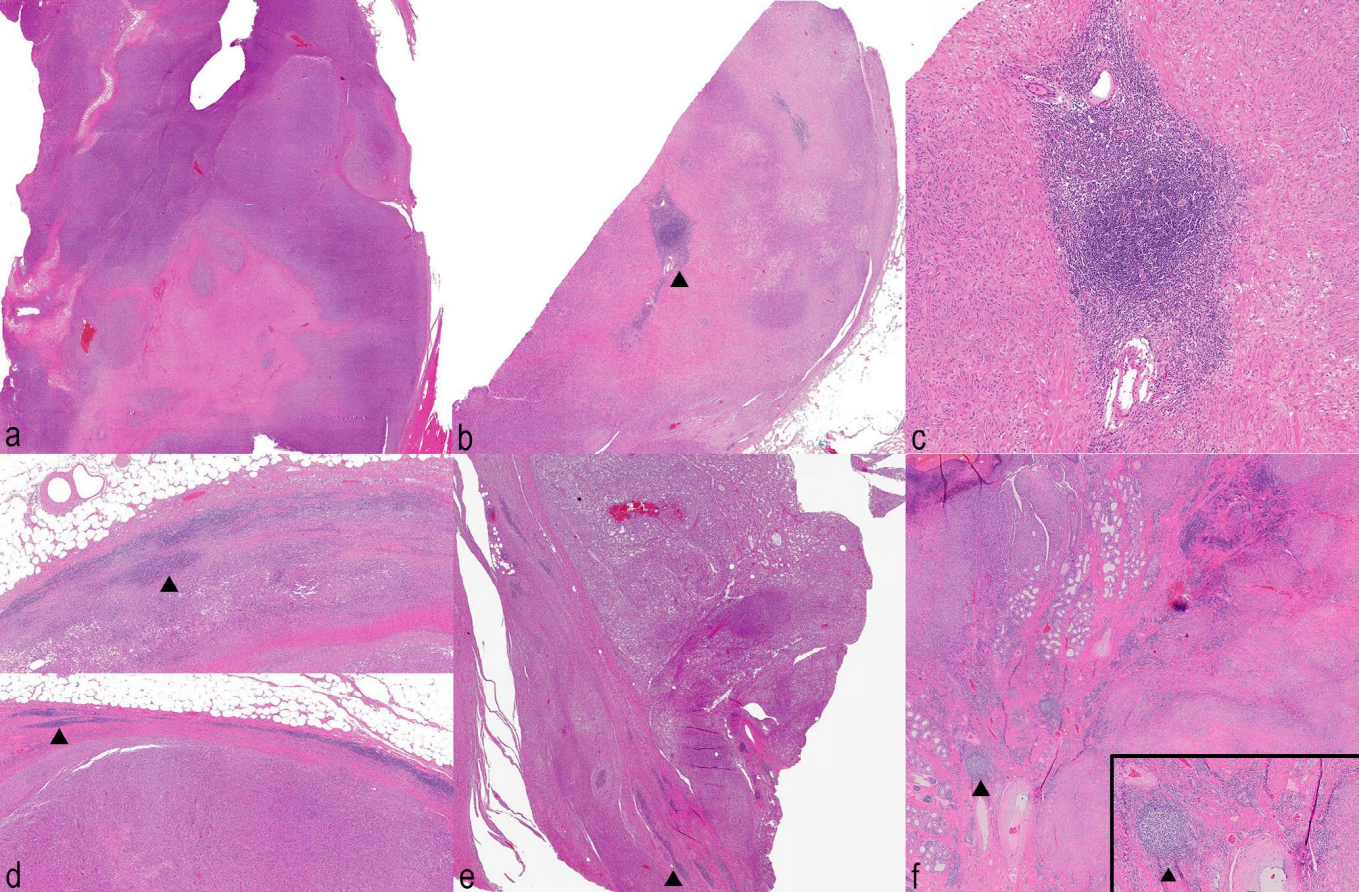
(a–f, H&E) Magnification: A, b, d–f 40x, C and inset of f 400x. Soft‐tissue sarcoma sections stained with haematoxylin and eosin. Physical slides were digitised using the Olympus VS120 Scanning Microscope. Still images were taken from the Olyvia desktop programme. Arrowheads indicate lymphoid aggregate in each image. (a) Sample with no aggregates. (b) Sample with intratumoral aggregate. (c) High magnification image of aggregate in b. (d) Top and bottom show sample with peripheral aggregates. (e) Sample with peripheral aggregates. (f) Sample with peripheral aggregates with morphology including germinal centre development (arrowhead; the inset shows this at higher magnification).

A search was performed in the electronic medical records of canine patients seen between the years 2019 and 2022 at the Colorado State University Diagnostic Laboratory to identify sarcomas with LAs. This population represents a subset of sarcomas containing LAs, as we specifically selected for large aggregates. Permission for use of collected tissues was given at the time of patient admission or sample submission. Specific verbiage on the patient intake form is: XXX is continually reviewing medical information to improve patient care. Therefore, the university may use information, excluding identifying data, for research, medical studies, publication, or teaching purposes. This may include information from the hospital visit, in the medical record, materials prepared by the university for its use, or related biologic materials destined for disposal (e.g., remaining blood or tissue collected for diagnostic purposes). The text of the pathologic description was searched for “lymph*” and “aggregate*” to find candidate cases for TLS‐containing tumours (where the asterisk is a wildcard). Candidate slides were screened microscopically for evidence of LAs. Sarcoma cases were included in the study if they contained at least four LAs of at least 100 cells each and adequate adjacent tumour tissue for harvest. Control samples included three STSs without LAs, and three normal or hyperplastic canine lymph nodes selected from the daily case stream in 2021. Rhabdomyosarcomas and leiomyosarcomas as well as plexus‐associated peripheral nerve sheath tumours were excluded. Examples of large LA containing sarcomas and aggregate‐free sarcomas are provided in Figure 1. All cases were reviewed and graded by an anatomic pathologist (PS) based on Dennis et al. [3].

### 2.4 Section Preparation for Laser Capture Microdissection

Formalin‐fixed paraffin‐embedded (FFPE) tissue blocks corresponding to the chosen case subset were retrieved from the archives. The 10 μm‐thick sections were cut using a Leica RM2245 Microtome onto polyethylene naphthalate (PEN) membrane slides (Fisher‐Scientific), dried for 24 h in a 37°C oven, deparaffinised and stained with haematoxylin and eosin using a procedure modified from Golubeva et al. [24]. Briefly, the sections were deparaffinised for 5 min in two sequential xylene baths, followed by sequential rehydration in 100%, 90%, 70%, 50% and 30% ethanol baths for 30 s each. Staining was carried out by placing slides for 2 min in Mayer's haematoxylin (Sigma), 1 min in Scott's tap water substitute, 5 s in eosin Y (Sigma), followed by a brief rinse in 100% ethanol. The slides were either cut immediately or frozen at −80°C until laser capture. Immediately prior to laser capture micodissection (LCM), the slides were dried in a dessicator for 60 min at room temperature. LCM was carried out in a Leica LMD7000 (Leica Microsystems). Aggregates were visualised at low magnification, and LCM was carried out at 100x magnification using a range of laser power settings, apertures and pulse rates adjusted to each tissue for efficient cutting. Multiple sections of lymphoid aggregate (LA) tissue and adjacent sarcoma tissue (aggregate associated sarcoma, AAS) were cut and pooled. Aggregate‐free sarcoma (SA) tissue and lymph nodes (LNs) were harvested via curls using the Leica RM2245 Microtome. The total area of tissue for RNA isolation ranged from 1.3 × 10^6^ μm [2] to 23.5 × 10^6^ μm [2]. The area of each tissue and RNA quantification is shown in Table S1. Tissue sections were harvested directly into 0.5 mL microcentrifuge caps containing 20 μL of lysis buffer from the nucleic acid isolation kit. Samples were stored in −80°C until RNA was extracted. Representative images of outlined areas to be cut by LCM and section inside caps are shown in Figure S1.

### 2.5 RNA Extraction and NanoString Analysis

RNA was isolated from the LCM tissue using a commercial kit (RecoverAll Total Nucleic Acid Isolation, Invitrogen). Because the samples had already been deparaffinised, that step was omitted during RNA isolation. RNA quality was evaluated on the Bioanalyzer (Agilent, Santa Clara, CA) at the Curie Institute, Paris, France, where the digital counting was performed. RNA copy counting was performed using the NanoString nCounter gene expression system (nanostring.com). Briefly, the optimal amount of total RNA (100–500 ng) was hybridised with the custom codeset in an overnight incubation at 65°C, followed by processing on the NanoString nCounter FLEX Analysis System. The quality of the RNA was judged based on concentration of RNA and the percentage of RNA fragments that are greater than 200 nucleotides in length (DV200) as determined by the Bioanalyzer instrument (Agilent, Santa Clara, CA). LCM area, RNA concentration as determined by the Qubit (ThermoFisher), and RNA quality are listed for each sample in Table S1. The 800 genes included in the nCounter Canine IO Panel (https://nanostring.com/products/ncounter‐assays‐panels/oncology/canine‐io/) are available online. The panel includes 20 housekeeping genes, six positive control probes and eight negative control probes.

### 2.6 Immunohistochemical Staining

Immunohistochemistry was performed on seven unique LA cases with large (> 700 cells), organised lymphoid aggregates. Six of the seven sarcoma sections were obtained from the same blocks from which the LCM was performed. One block was consumed in the LCM sectioning and was not included in the IHC. An additional section from a tumour block used for LCM was included to provide additional LA examples. IHC was performed on serial 5 μm‐thick, paraffin‐embedded, formalin‐fixed (FFPE) tissue sections with labelling for CD3, CD20, MUM1 and MECA79 (peripheral node addressin, PNAd). Deparaffinisation, antigen retrieval, IHC and counterstaining were performed on a Leica Bond automated staining system. The antibodies used and procedure notes are shown in Table 1.

**TABLE 1:** Summary of antibodies and procedures used for mmunostaining.

Target	Host species	Supplier	Clone	Working concentration (μg/mL)	Antigen retrevial*	Antibody incubation period	Chromogen**	Positive control
High endothelial venule marker (PNAD)	Rat	ThermoFisher	MECA79	10	ER1	30 min	DAB	Canine lymph node
CD20 pan B‐cell	Rabbit	SinoBio	Poly	0.5	ER1	30 min	DAB	Canine lymph node
CD3 pan T‐cell	Mouse	Leica	LN10	Ready to use	ER1	30 min	DAB	Canine lymph node
MUM1 plasma cells	Mouse	ThermoFisher	MUMp1	100 fold dilution	ER2	30 min	DAB	Canine lymph node

* Leica BondRx Bond Epitope Retrevial Method (95°C, 30 min).

** Leica Bond Polymer Refine (3,3'diaminobenzidine).

### 2.7 Visiopharm Digital Analysis

IHC slides were digitised with an Olympus VS120 Scanning Microscope (20X). The whole slide scans were uploaded and analysed using Visiopharm Quantitative Digital Pathology software. Haematoxylin only slides of each sample were analysed using the Visiopharm software nuclei detection app to determine which of the LAs contained at least 700 lymphoid cells and were larger than 60 000 μm [2] as defined by Italiano et al. [14]. We chose this size to evaluate the most likely aggregates to have mTLS characteristics. The aggregates that met the criteria were then identified in the serial immunohistochemical sections. A section of tumour surrounding the aggregate was chosen as the AAS tissue for comparison. The Visiopharm nuclei detection application was then used to quantify CD3 + CD20+ and MUM1‐positive cell populations inside of the LA and in adjacent AAS tissue. The Visiopharm cell counts were compared with visual counts of scanned CD3 images, as shown in Figure S2, to ensure correlation with visual counts of positive cells. The application allowed us to find the density of each cell population in the selected area using the nuclei detection programme. HEVs were visually counted from the scanned slides as the number of individual vascular structures that expressed MECA79.

### 2.8 Data Analysis

The RCC files provided by NanoString were analysed using the nSolver software version 4.0. This software allows for raw count normalisation to the expression of housekeeping genes and for background correction. For background correction, the geometric means +/− 2 SD of the negative controls were subtracted from the counts obtained for each gene. The raw data were analysed for differential expression (fold change and adjusted *p* value) and unsupervised hierarchical clustering of all genes using the Advanced Analysis available on the nSolver software with a false discovery rate of < 0.05 shown in Figure S3. Agglomerative hierarchical clustering was then used to produce a heat map for a list of “TLS‐rich” genes, curated from the literature, shown in Table 2. The heat map was generated by Euclidean distance and mean distance. No QC flags were found on any of the samples. Numbers of positive cells on IHC (B or T cells) after Visiopharm analysis within LA was compared to numbers AAS with a paired *t*‐test.

**TABLE 2:** RNA levels of 27 immune cell genes between LA and AAS.

	Gene name	Function	LA versus AAS fold change	*p*
B cells and plasma cells	CD20	B‐cell lymphocyte antigen	13.8	**0.0003**
	CD19	B‐cell lymphocyte antigen	17.4	**0.0001**
	CD22	B‐cell receptor	6.3	**< 0.0001**
	BLK	B lymphoid tyrosine kinase	9.1	**0.0002**
	SPIB	Spi‐B transcription factor, activate JAK/STAT pathway	7.3	**0.0008**
	IgGH	Immunoglobulin heavy chain constant gamma	5.1	**0.019**
	IgGM	Immunoglobulin heavy chain constant mu	4.9	**0.0012**
	BCL6	Transcription factor for regulation of T follicular helper cells proliferation	0.74	0.0745
	IRF4	Marker for plasma cells (transcription factor)	2.58	**0.00422**
T cells	CD3	T‐cell surface glycoprotein	2.3	**0.043**
	CD8	T‐cell surface glycoprotein on CD8 T cells	1.7	0.245
	CD4	T‐cell surface glycoprotein on CD4 T cells	1.8	0.165
	CXCR5	Enables T cells to migrate to lymph nodes and to TLS in tumours	5.8	**< 0.0001**
	TBX21	TH1 T cell–specific transcription factor	1.1	0.826
	CCR7	Controls migration of immune cells to secondary lymphoid organs	6.7	**< 0.0001**
	PD‐1	Programmed cell death protein‐1	0.724	0.786
	FOXP3	Transcription factor expressed by CD4+ T cells	2.0	0.0867
Dendritic cells	CD83	Cell surface marker for fully mature dendritic cells	1.4	**0.0549**
	CD86	Cell surface markers on antigen‐presenting cells	0.74	0.325
	CCL19	Chemokine that regulates the induction of T‐cell activation	3.68	**0.0209**
	CCL21	Chemokine that is involved in lymphatic transmigration	6.87	**0.0209**
Formation of TLS	LTA	Lymphotoxin alpha, tumour necrosis factor	2.73	**0.0451**
	LTB	Lymphotoxin beta, tumour necrosis factor	6.45	**0.00142**
	LTBR	Receptor for LTB, involved in apoptosis and cytokine release	0.46	**0.00242**
	VCAM1	Vascular cell adhesion protein 1	1.4	0.450
	ICAM1	Intracellular adhesion protein 1	0.9	0.711

	CCL19	Chemokine ligand, promote infiltration of DC and T cells into tumour	**3.68**	**0.0209**
	CCL21	Chemokine expressed by DC and T cells for antitumour responses	**6.87**	**0.0209**
CXCL13	Chemokine that promotes chemotaxis of B cells, germinal centre formation	2.68	**0.0924**
Infiltrating cells	IL17RA	Interleukin proinflammatory cytokine	1.0	0.889
	NCR1	Recognition and destruction of tumour cells	1.36	0.54

*Note*: Bolded *p*‐values are considered statistically significant or represent important molecules that show a trend toward significance.

## 3 Results

### 3.1 Retrospective Study for Small and Large Lymphoid Aggregate Prevalence

Of the 100 tumours that were analysed histologically, at least one section of 30 of the tumours contained at least one small aggregate (prevalence of 30%). Of the same 100 tumours, at least one section of 12 of the tumours contained at least one large aggregate (12% prevalence). Table S2 details the signalment, histologic grade, total slides per tumour and number of aggregates within the tumour.

### 3.2 RNA Expression of Large Lymphoid Aggregates

**FIGURE 2:**
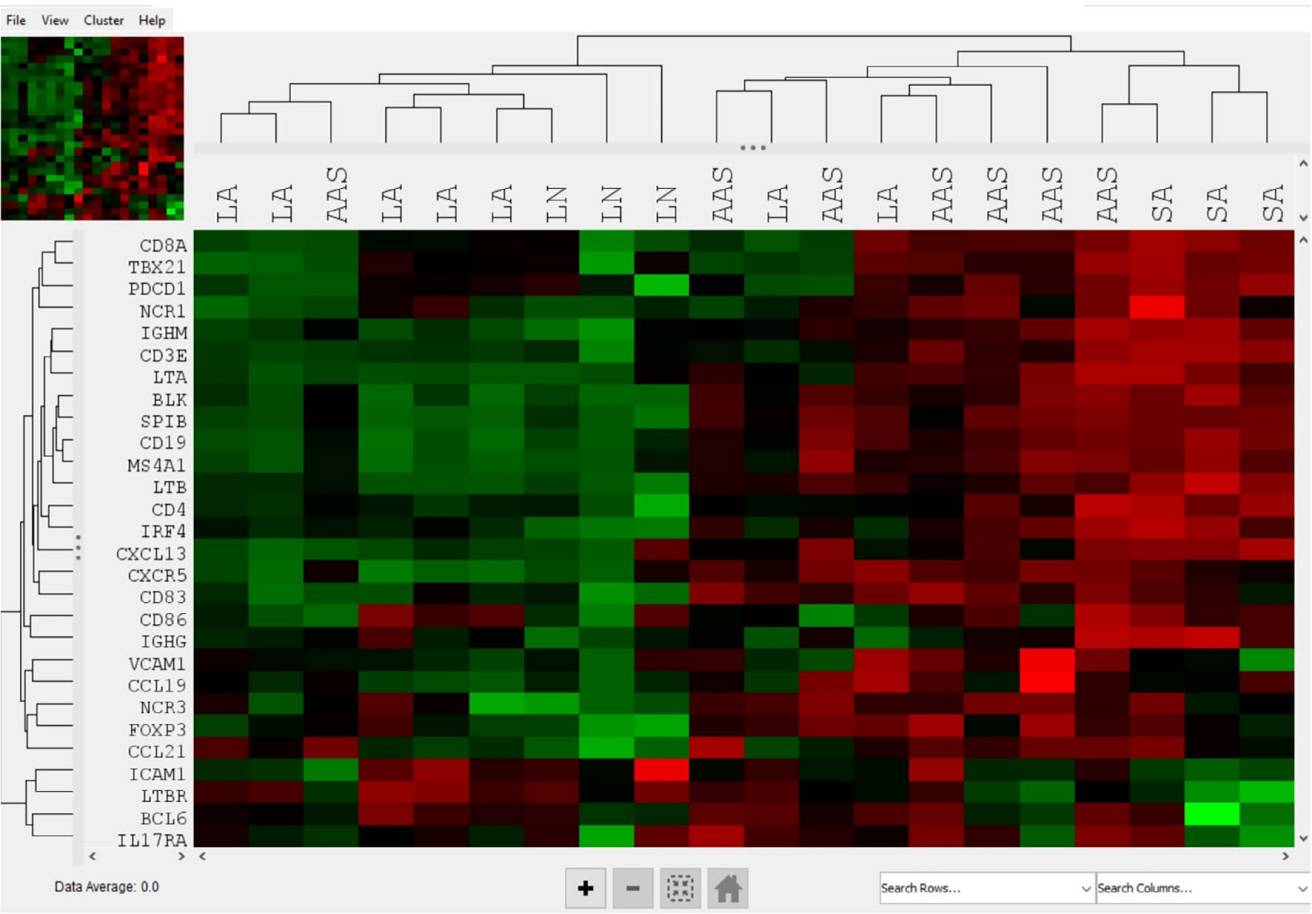
Clustering analysis of the TLS‐specific canine IO genes using RNA copy number to identify trends in clustering. LA = lymphoid aggregate tissue, AAS = aggregate‐adjacent sarcoma tissue, LN = lymph node, SA = sarcoma without lymphoid aggregates.

A heat map was created using a curated set of genes common in TLS, as shown in Figure 2 [25]. This heat map places four AAS with the SA tissues, and only two AAS tissues with the LA and LN tissues. To describe how each of the specific immune cell populations differed within LA and AAS, selected individual genes contained in the IO panel for each cell type were chosen from the differential expression analysis. Table 2 presents the results of gene expression from genes extracted from the literature that are thought to be important in TLS generation and function. Genes that are indicators of B cells and B‐cell functions were all more highly expressed in LA compared to AAS. These genes include CD20 (13.8 fold, *p* = 0.0003), CD19 (17.4 fold, *p* = 0.0001), BLK (9.1 fold, *p* = 0.0002), SPIB (7.3 fold, *p* = 0.0008), IgHG (5.1 fold, *p* = 0.019) and IgMG (2.3 fold, *p* = 0.043). These values show that the B cells are concentrated inside of the LAs when compared with the surrounding tissue. Additional genes with significantly increased expression in LA versus AAS include CXCR5 (fold change 5.8, *p* = < 0.0001), CCR7 (6.7 fold, *p* < 0.0001), CD83 (1.4 fold, *p* = 0.0549), CCL19 (fold change 3.86, *p* = 0.0209), CCL21 (fold change, *p* = 0.0209), lymphotoxin alpha (LTA) (fold change 2.73, *p* = 0.0451), lymphotoxin beta (LTB) (fold change 6.45, *p* = 0.00142) and lymphotoxin beta receptor (LTBR) (fold change 0.46, *p* = 0.002).

### 3.3 Immunohistochemical Evaluation of Immune Cell Markers

**FIGURE 3:**
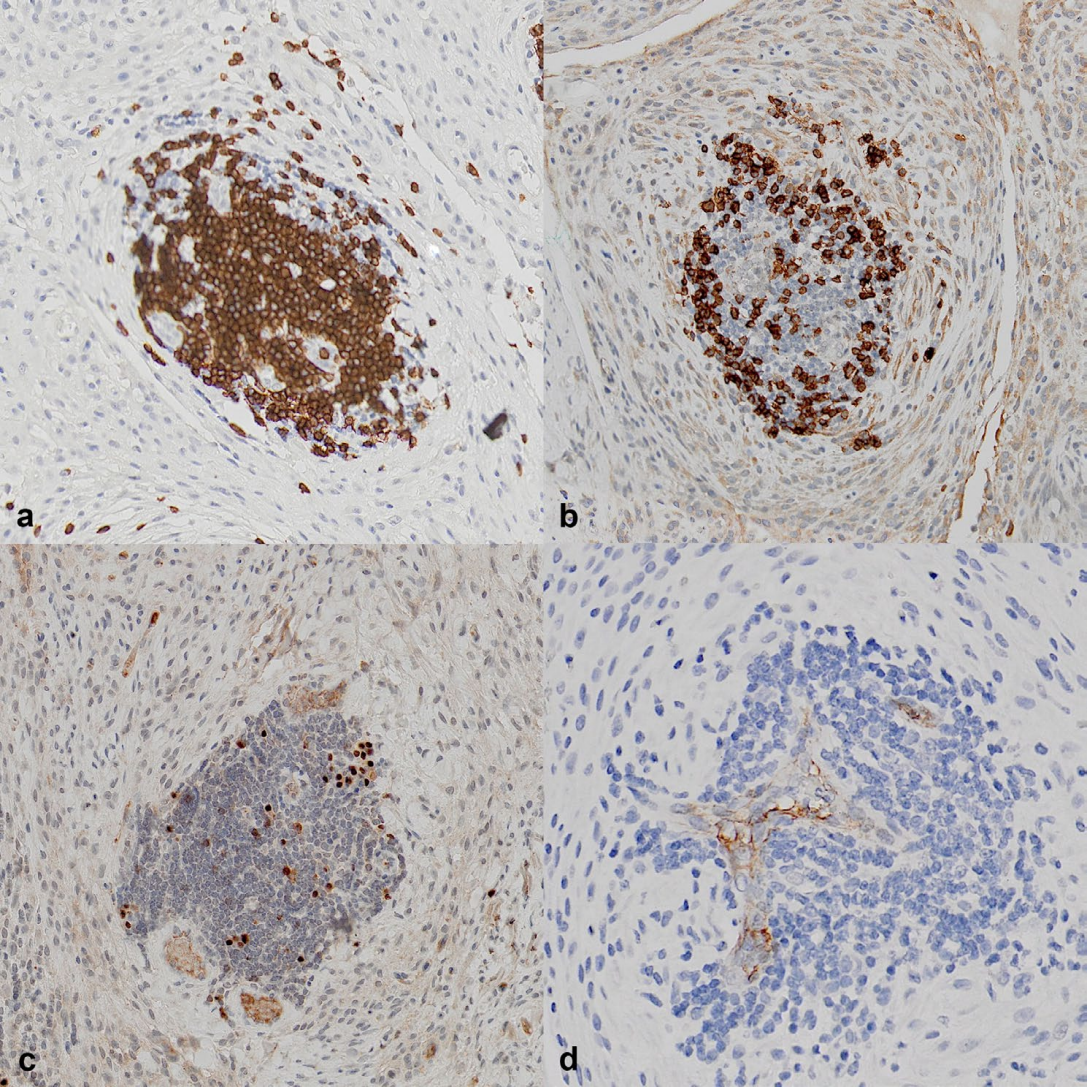
(a–d) Serial slides represent one sarcoma‐associated lymphoid aggregate prepared with CD3, CD20, MUM‐1 and Meca79 immunohistochemistry (DAB chromagen), all magnifications = 400x. (a) Anti‐CD3 showing distribution of T cells in a sarcoma‐associated lymphoid aggregate. (b) Anti‐CD20 showing the distribution of B cells in the same aggregate. (c) Anti‐Mum1 showing plasma cell distribution. (d) Anti‐Meca79 localises peripheral lymph node addressin to small blood vessels within the aggregate.

Figure 3 depicts the reaction pattern for each antibody in a single LA and is representative of the pattern seen with all other aggregates evaluated by immunohistochemistry. B cells are concentrated inside of LA and are far less frequent in AAS. T cells are less densely distributed inside of the LA and are present more commonly in AAS. Immunohistochemistry positively identified plasma cells and HEVs. MUM1‐ and MECA79‐positive cells were identified in all tumours with LAs. However, no MUM1‐ or MECA79‐positive cells were found in the tumours lacking LAs. Cell counts quantified with Visiopharm are summarised in Table 3. The average density of the B cells inside of the LA was 366 nuclei/60000 μm^2^ and 5.4 nuclei/60000 μm^2^ in AAS, a fold difference of 67.8. The percent positivity of B cells inside LA was 50.37% and 1.11% in AAS, a 45.4 fold difference. The T‐cell density and percent positivity in LA versus AAS were 256 nuclei/60000 μm^2^ versus 82 nuclei/60000 μm^2^ (3.12 fold) and 37.74% versus 22.94% (1.64 fold). The number of HEVs ranged from 3 to 43, based on visual counting of the entire scanned section. In the three negative control sarcomas, there were no HEVs.

**TABLE 3:** Statistical findings of IHC images * = *p* < 0.05 in paired *t*‐test.

Case	Density TLS (Nuclei/60 000 μm [2])		Density tumour (Nuclei/60000 μm^2^)		%Positive TLS		%Positive tumour	
	CD3	CD20	CD3	CD20	CD3	CD20	CD3	CD20
1	420	258	120	0.504	62.07	33.23	27.6	0.08
2	246	600	48	4.98	35.23	78.55	16.5	0.93
3	138	258	66	9.6	26.17	41.69	19.59	2.17
4	216	330	126	13.2	32.3	48.86	36.56	2.82
5	198	384	60	0.204	28.08	54.15	28.96	0.12
6–1	210	312	144	7.8	29.32	41.02	23.06	1.36
6–2	366	420	42	1.74	51.04	55.05	11.3	0.28
Mean	256*	366*	82	5.4	37.74*	50.37*	22.94	1.11

## 4 Discussion

TLSs are important structures in many human cancers and have generated intense research since the seminal paper in 2008 [17]. Their presence improves the prognosis of most tumours, and research is ongoing to investigate inducing them in tumours to promote an antitumour response [26]. Previous research in humans have defined TLS based on size criteria as well as by containing dense B‐cell populations, T‐cell populations, plasma cells and HEVs although their definition is not standardised [13, 15]. Work in the field is ongoing to provide standardisation of TLS definition and enumeration (Marie‐Caroline Dieu‐Nosjean, personal communication). Using the above criteria, we determined that the LAs in canine STSs meet key genetic and immunologic criteria of TLS. Our long‐term goal is to determine the role that these aggregates play in prognosis and therapy. In human medicine, there is a push to induce these structures in tumour to provide a localised antitumour response [27].

The NanoString data of AAS grouped closely with LN samples in the agglomerative clustering using a TLS‐rich gene set, closely aligning them with this secondary lymphoid structure, a classic example of immune organ composition and function [28]. In our immunohistochemical analysis, we found two other important features of TLS, HEV and plasma cells, enriched in LAs.

Both B cells and plasma cells have recently been shown to be key players in the antitumour immune response, and their presence enhances prognosis in several human tumours [11, 20, 30, 32, 35]. B cells act as antigen‐presenting cells (APCs), produce antitumoral antibodies for complement mediated lysis or antibody‐dependent cytotoxicity, and secrete cytokines such as TNF, IL‐2, IL‐6 and IFNγ which attract immune cells including T cells [16, 20, 26, 29, 30, 31]. Germinal centres are responsible for producing affinity matured and class switched B cells and are indicative of mature TLS with maximal engagement of the humoral arm of the immune system [32]. We saw RNA evidence of class switching, as we saw equal amounts of IgM and IgG heavy chain RNA. Plasma cells that are created inside of the TLS can produce antibodies that target‐specific tumour‐associated antigens [33].

Markers and chemokines considered important for TLS definition and function were found in the NanoString gene expression analysis. These include T‐cell subsets, and chemokines important for immune cell migration and function. CD4+ T cells secrete proinflammatory cytokines and chemokines to recruit other immune cells, and can kill tumour cells using an MHC II‐dependent mechanism [34]. CD8+ T cells once activated for a specific antigen release granzymes that induce apoptosis and can kill the tumour cells [35].

The RNA expression of some of these genes, such as LTA, LTB, CCL19, CCL21 and CXCR5, provides additional support that these aggregates are TLS. Lymphotoxin beta (LTB) is an agonist in the lymphotoxin beta receptor (LTBR) pathway which induces the expression of chemokines and adhesion molecules including, CLL19, CCL21 and CXCL13, and is important in the differentiation of precursor cells into follicular dendritic cells (FDCs), the initial clustering of lymphoid cells, and the development of HEV [36]. CCL21 is a chemokine produced by HEVs, lymphatic endothelial cells and stromal cells in T‐cell areas of LNs. CCL21 chemoattracts T cells and dendritic cells (DC) and is crucial for the T‐cell activation in LNs. For TLS, it is thought that APCs, such as DC, and T cells, are both attracted to the aggregates via CCL21 and once within the aggregates the T cells are activated against antitumoral antigens [37]. CXCL13 and CXCR5 is a chemokine receptor pair that has been shown to be important in TLS formation. CXCL13 is a B‐cell chemoattractant and is expressed by CD4+ T cells. When CXCL13 secreting T cells infiltrate a tumour they can attract B cells expressing the CXCR5 receptor and cause aggregates of B cells to form, therefore inducing the formation of a TLS [26]. The increased expression of CXCR5 inside the aggregates also supports the presence of the agonist‐receptor pair since it is the receptor of CXCL13. CXCL13 was increased inside the aggregates and approached statistical significance (*p* = 0.09). The fact that CXCL13 was not significantly increased in the aggregates may be because there are also T cells in the diffuse portion of the tumour. These may play a role in attracting B cells to the tumour itself, despite the fact that they are not in aggregates.

HEVs are specialised blood vessels that allow for the passage of naive lymphocytes from the bloodstream into the LNs and, by extension, predicted to be important in the infiltration of TLS by lymphocytes [38]. In human cancers, tumour‐HEVs (TU‐HEV) have been reported in T‐cell and DC‐rich areas and also in B cell–rich areas, and their presence is implicated in the formation of immune‐active TLS structures [39]. The LTB signalling pathway, namely LTA, LTB and LTBR, has been linked to the development of HEV in LNs [36, 40]. HEVs also express high levels of the ligand L‐selectin which is a classic homing receptor for the T and B lymphocytes [40]. In the NanoString data, we detected a 5‐fold increase of LTB and a 3.6‐fold increase of L‐selectin in the aggregates compared with the adjacent sarcoma tissue, suggesting that the stimulus for HEV formation is within the tumour. The identification of the HEV via MECA79 antibody and upregulation of the L‐selectin and LTB in aggregates leads to the conclusion that there is HEV present supporting the identification of the aggregates as TLS.

We have found that 30% of tumours contain at least one small aggregate of lymphocytes (> 50 cells), and 12% contain at least one large aggregate (> 700 cells). In humans, the prevalence of LAs in sarcomas has been reported to be about 18% [13]. The presence of TLS in canine sarcomas may have implications for prognosis and may eventually lead to additional investigation into immunotherapeutic strategies. Inflammatory aggregates are not part of the current scheme for grading of canine STSs, but our work indicates that the presence of histologic lymphoid aggregates is a feature that may warrant investigation for future diagnostic reporting. Canine sarcomas may be important in translational medicine and is already described as a natural model for human sarcomas [41].

This study is limited by the small sample size of RNA‐ and IHC‐analysed sarcomas, and the fact that the RNA was extracted from FFPE tissue. Another limitation of this study was the lack of identification of immunohistochemical FDCs, as we were unable to find an antibody that did not also react with B cells. FDC are strong indicators of TLS in humans and identifying them would have provided further evidence that they are TLS. Additionally, we do not provide follow‐up information in this study, as prognostic research is ongoing. Additionally, the nature of a retrospective study prohibits evaluation of the entire tumour for the presence of TLS, potentially falsely decreasing the prevalence estimate of LA.

We have confirmed the presence of TLS in canine sarcomas based on size and the dense B‐cell populations with HEV, T cells and plasma cells present. This was further correlated with the presence of LTB, CCL21 and CXCR5 which are important signalling molecules in the formation of TLS and the recruitment of immune cells. Although this initial characterisation of TLS in canine sarcomas is promising, much more can be investigated to gain a larger picture of the aggregates and their function. To look more at the composition of the TLS, identification of FDCs could be performed using appropriately validated antibody, and the composition of T‐cell types would be helpful (CD8, CD4, Treg, activated T cells, exhausted T cells). CD4 and CD8 quantification will require frozen sections of sarcomas and potentially flow cytometry of disaggregated tumours.

## Ethics Statement

Permission was granted for use of tissue sections at the time of admission to the CSU Veterinary Teaching Hospital. Specific verbiage in the patient intake form, which clients sign on admission: CSU is continually reviewing medical information to improve patient care. Therefore, the university may use information, excluding identifying data, for research, medical studies, publication or teaching purposes. This may include information from the hospital visit, in the medical record, materials prepared by the university for its use, or related biologic materials destined for disposal (e.g., remaining blood or tissue collected for diagnostic purposes).

## Conflicts of Interest

The authors declare no conflicts of interest.

## Supporting information


**Figure S1.** Representative images of LA and AAS laser dissection. A. Case example 1. The turquoise lines outline LA tissue, and the fuchsia lines outline adjacent sarcoma tissue. B. Case example 2. The turquoise lines outline TLS tissue, the black lines outline adjacent sarcoma tissue. C and D. Dissected sections of AAS and LA tissue, respectively, in the collection tube caps.

**Figure S2.** To verify that the APP developed in Visiopharm was accurate in counting positive cells, screenshots of selected lymphoid aggregates were counted visually for positive cells per aggregate and compared with the Visiopharm APP counting of the same aggregate. The aggregate was divided into sections and each section was counted visually. The total numbers from each section were calculated. A total of 10 aggregates from five sections were evaluated. A Bland–Altman plot for differences and correlation (Pearson) were performed between manual counting and Visiopharm counting (Graphpad prism). A. Examples of subdivided aggregate counted for CD3 positive cells and B. the same aggregate coded by the Visiopharm APP (red = positive, blue = negative). C. Correlation of manual versus Visiopharm data.

**Figure S3.** Unsupervised hierarchical clustering of the four groups: LN, LA, AAS and SA.

**Table S1.** Summary of case data, LCM area and RNA parameters.

**Table S2.** Summary of case data and TLS prevalence.

## Data Availability

The data that support the findings of this study are available from the corresponding author upon reasonable request.
