# Supplementary material for: Lymphoid Aggregates in Canine Cutaneous and Subcutaneous Sarcomas: Immunohistochemical and Gene Expression Evidence for Tertiary Lymphoid Structures
Source: Vet Comp Oncol. 2024 Oct 27;23(1):10–9. doi: 10.1111/vco.13020 (PMC11830466; doi:10.1111/vco.13020)
Supplement: Supplementary file 1 — Figure S1. Representative images of LA and AAS laser dissection. A. Case example 1. The turquoise lines outline LA tissue, and the fuchsia lines outline adjacent sarcoma tissue. B. Case example 2. The turquoise lines outline TLS tissue, the black lines outline adjacent sarcoma tissue. C and D. Dissected sections of AAS and LA tissue, respectively, in the collection tube caps. [file VCO-23-10-s002.docx]

Figure S1

Representative images of LCM

Images of software outlines before LCM cuts

**A**


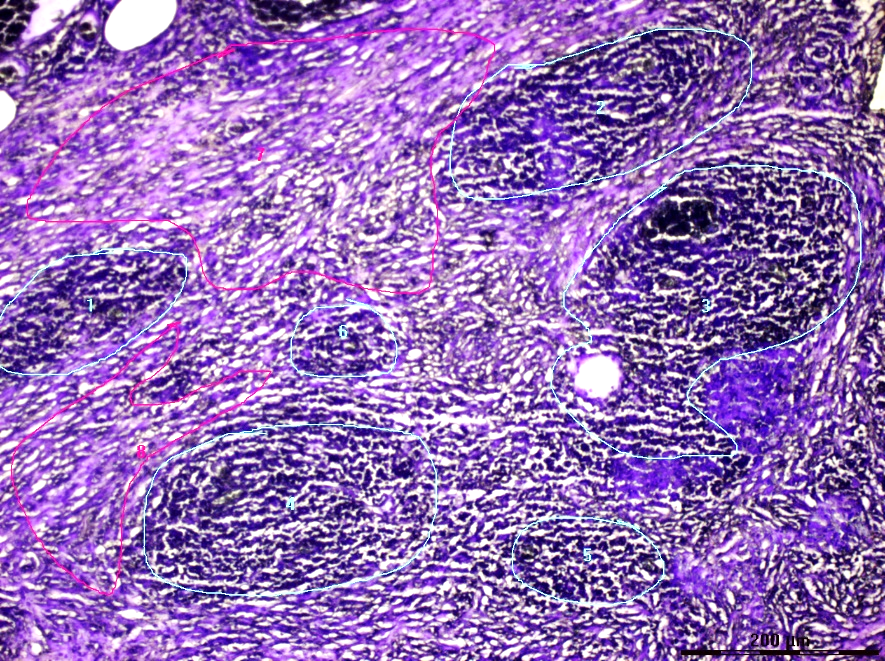


**B**

A. Case example 1. The white lines outline TLS tissue, the fuchsia lines outline adjacent sarcoma tissue


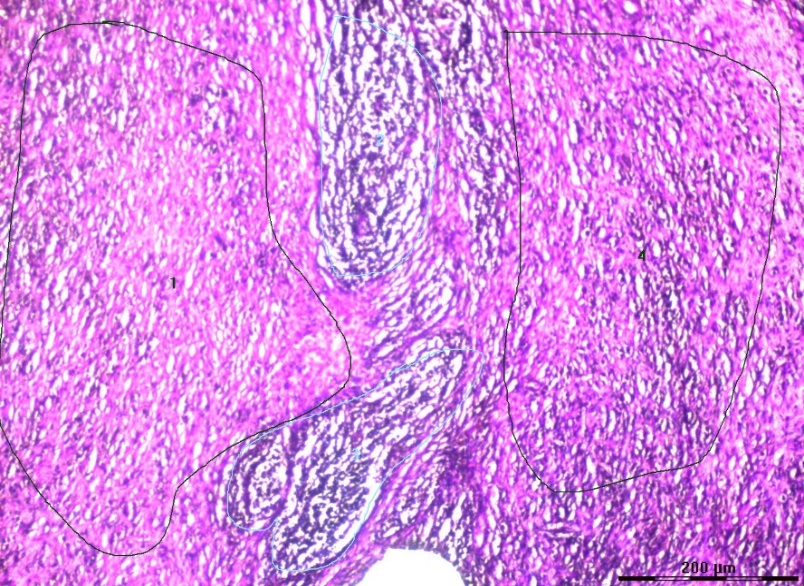


B. Case example 2. The white lines outline TLS tissue, the black lines outline adjacent sarcoma tissue

LMD cuts in the tube caps


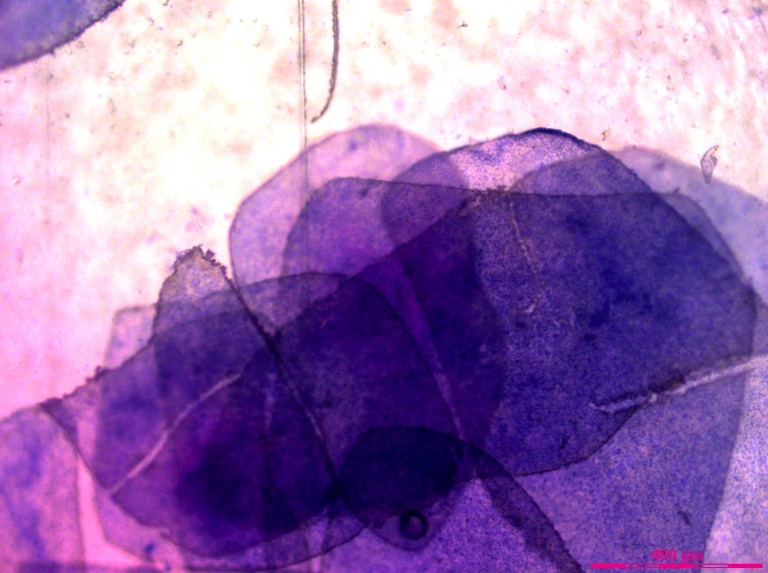

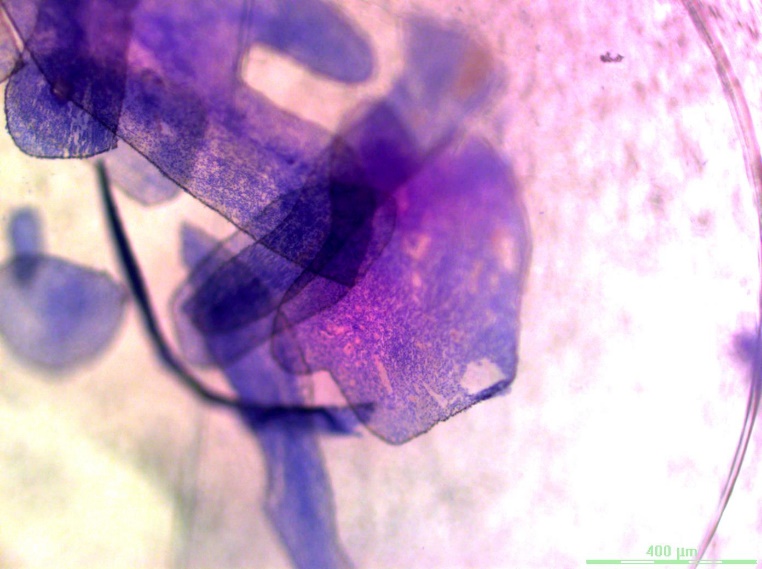


**D**

**C**

D. LA tissue sections in lysis buffer in the tube cap

C. AAS tissue sections in lysis buffer in the tube cap
