# Supplementary material for: Lymphoid Aggregates in Canine Cutaneous and Subcutaneous Sarcomas: Immunohistochemical and Gene Expression Evidence for Tertiary Lymphoid Structures
Source: Vet Comp Oncol. 2024 Oct 27;23(1):10–9. doi: 10.1111/vco.13020 (PMC11830466; doi:10.1111/vco.13020)
Supplement: Supplementary file 2 — Figure S2. To verify that the APP developed in Visiopharm was accurate in counting positive cells, screenshots of selected lymphoid aggregates were counted visually for positive cells per aggregate and compared with the Visiopharm APP counting of the same aggregate. The aggregate was divided into sections and each section was counted visually. The total numbers from each section were calculated. A total of 10 aggregates from five sections were evaluated. A Bland–Altman plot for differences and correlation (Pearson) were performed between manual counting and Visiopharm counting (Graphpad prism). A. Examples of subdivided aggregate counted for CD3 positive cells and B. the same aggregate coded by the Visiopharm APP (red = positive, blue = negative). C. Correlation of manual versus Visiopharm data. [file VCO-23-10-s001.docx]

FIGURE S2

Verification of visiopharm counting of CD3 positive cells

To verify that the APP developed in visiopharm was accurate in counting positive cells, screenshots of selected lymphoid aggregates were counted visually for positive cells per aggregate and compared with the visiopharm APP counting of the same aggregate. The aggregate was divided into sections and each section was counted visually. The total numbers from each section were calculated. A total of 10 aggregates from 5 sections were evaluated. A bland-altman plot for differences and correlation (Pearson) were performed between manual counting and visiopharm counting (Graphpad prism).

Examples of a subdivided aggregate counted for CD3 positive cells, and the same aggregate coded by the visiopharm app (red = positive, blue = negative)


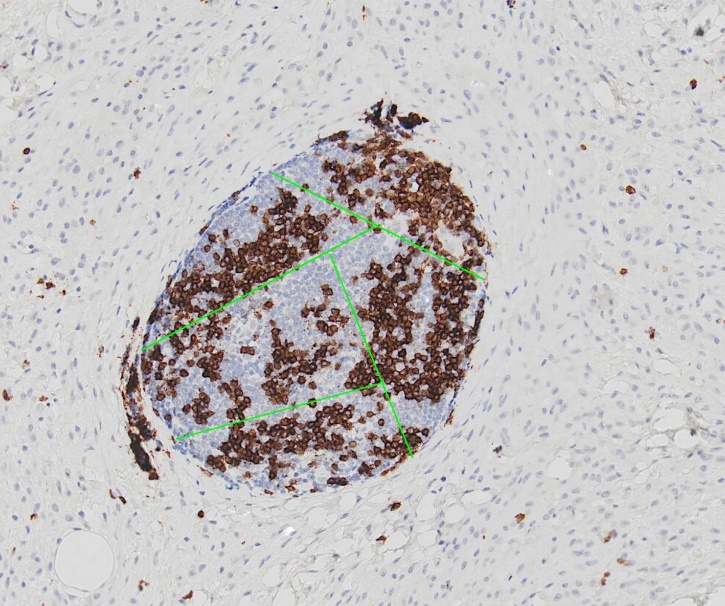

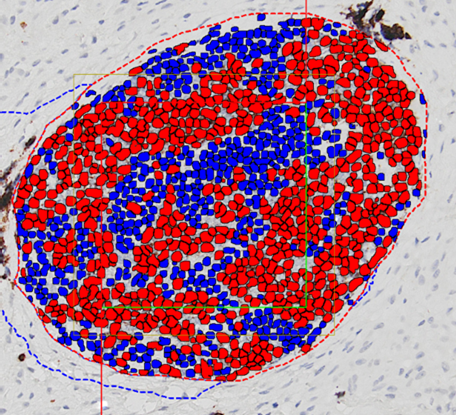


**B**

**A**

**C**

| Pearson r |  |
| --- | --- |
| r | 0.9963 |
| 95% confidence interval | 0.9840 to 0.9992 |
| R squared | 0.9927 |
|  |  |
| P value |  |
| P (two-tailed) | <0.0001 |
| P value summary | **** |
| Significant? (alpha = 0.05) | Yes |
|  |  |
| Number of XY Pairs | 10 |

Bland altman

| Bias | 57.15 |
| --- | --- |
| SD of bias | 91.82 |
| 95% Limits of Agreement |  |
| From | -122.8 |
| To | 237.1 |
