# Supplementary figures and images for: Lymphoid Aggregates in Canine Cutaneous and Subcutaneous Sarcomas: Immunohistochemical and Gene Expression Evidence for Tertiary Lymphoid Structures
Source: Vet Comp Oncol. 2024 Oct 27;23(1):10–9. doi: 10.1111/vco.13020 (PMC11830466; doi:10.1111/vco.13020)

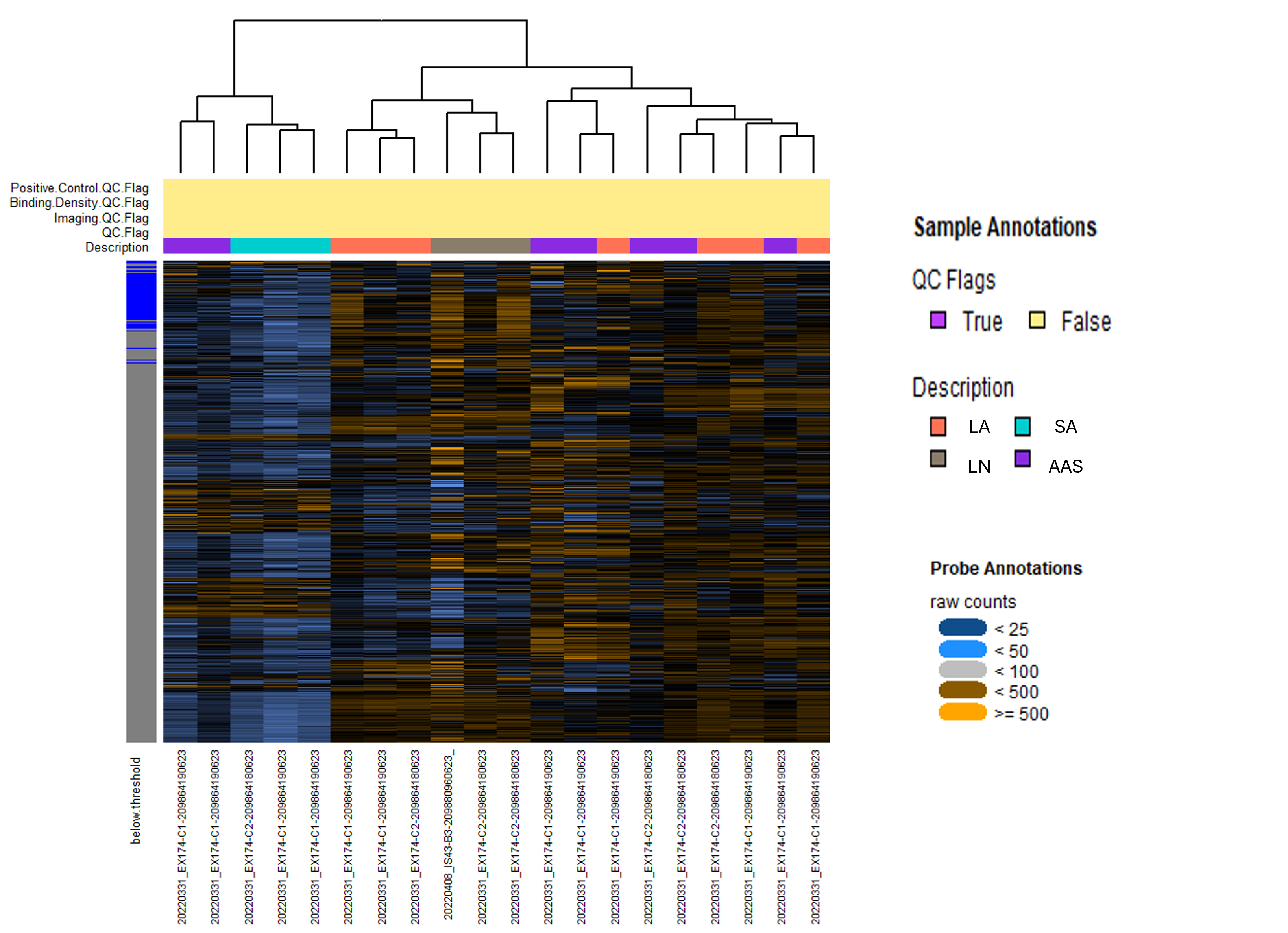

Supplement: Supplementary file 3 — Figure S3. Unsupervised hierarchical clustering of the four groups: LN, LA, AAS and SA. [file VCO-23-10-s005.tif]
