# Supplementary material for: Lymphoid Aggregates in Canine Cutaneous and Subcutaneous Sarcomas: Immunohistochemical and Gene Expression Evidence for Tertiary Lymphoid Structures
Source: Vet Comp Oncol. 2024 Oct 27;23(1):10–9. doi: 10.1111/vco.13020 (PMC11830466; doi:10.1111/vco.13020)
Supplement: Supplementary file 4 — Table S1. Summary of case data, LCM area and RNA parameters. [file VCO-23-10-s004.docx]

| Case | Type | Grade | Area LCM TLS (x10^6^ μM^2^) | Area LCM SA (x10^6^ μM^2^) | RNA TLS/DV200  (ng/ μl) | RNA SA/DV200  (ng/ μl) | IHC | Signalment |
| --- | --- | --- | --- | --- | --- | --- | --- | --- |
|  |  |  |  |  |  |  |  |  |
| 798-4 | SA-TLS | 2 | ND | ND | ND | ND | yes | 7 yr MC Labrador |
| 745-5 | SA-TLS | 2 | 19.5 | 18.6 | 25.8/61 | 13.0/61 | yes | 9 yr FS shepherd mix |
| 798-5 | SA-TLS | 2 | 16.8 | 17.8 | 30.6/70 | 27/60 | yes | 7 yr MC Labrador |
| 270-1 | SA-TLS | 2 | ND* | 9.6 | 76.2/61 | 24.6/57 | No^ | 15 yo chihuahua mix |
| 888-4 | SA-TLS | 2 | 6.3 | 7.7 | 7.2/64 | 18.1/64 | yes | 9 yr FS MBD^+^ |
| 669-1 | SA-TLS | 1 | 12.6 | 28.9 | 22.4/62 | 34/77 | yes | 11 yr MC BDF** |
| 846-3 | SA-TLS | 2 | 9.3 | 23.1 | 23/61 | 23.8/61 | yes | FS chihuahua |
| 587-7 | SA-TLS | 2 | NR* | NR* | 5.8/69 | 16.7/76 | yes | 12 yr FS Catahoula |
| 777 | LN | NA | curls | NA | 800/72 |  | NA | 9 yr FS Beagle |
| 736 | LN | NA | curls | NA | 8.84/72 |  | NA | FS Aus Cattle dog |
| 930 | LN | NA | curls | NA | 168.8/13 |  | NA | 12 yr FS Maltese |
| 216 | Non TLS sarcoma | 2 | curls | NA |  | 21.4/80 | NA | 12 yr FS Labrador |
| 736 | Non TLS sarcoma | 2 | curls | NA |  | 42.8/77 | NA | 10 yr MC Lab mix |
| 771 | Non TLS sarcoma | 2 | curls | NA |  | 118/59 | NA | 9 yr FS Shiba Inu |

Table S1

*lymphoid tissue extracted directly from the block

**Bouvier de Flandres

^Tissue consumed for RNA

^+^MBD = Mixed breed dog

*NR = not recorded, LMD software error
